# Comparative Study on Jejunal Immunity and Microbial Composition of Growing-Period Tibetan Pigs and Duroc × (Landrace × Yorkshire) Pigs

**DOI:** 10.3389/fvets.2022.890585

**Published:** 2022-04-25

**Authors:** Yuting Yang, Yongxiang Li, Yanggang Xie, Shiyan Qiao, Lijie Yang, Hongbin Pan

**Affiliations:** ^1^Yunnan Provincial Key Laboratory of Animal Nutrition and Feed Science, Faculty of Animal Science and Technology, Yunnan Agricultural University, Kunming, China; ^2^State Key Laboratory of Animal Nutrition, Ministry of Agriculture Feed Industry Centre, China Agricultural University, Beijing, China

**Keywords:** Tibetan pigs, DLY pigs, jejunum, immune, microbial composition

## Abstract

The gut microbiota plays vital roles in metabolizing nutrient, maintaining the intestinal epithelial barrier but also in modulating immunity. Host genetics and the pig breed are implicated in shaping gut microbiota. Tibetan pig is a unique native Chinese breed and has evolved to manifest a strong disease resistance. However, the immunity and microbiota of growing Tibetan (TP) pigs were still rarely understood. The jejunal immunity phenotype and microbial composition of TP and Duroc × (Landrace × Yorkshire) (DLY) pigs were explored through immunohistochemistry and 16S rRNA sequencing. Higher scores of clusters of differentiation 4 (CD4+) and Toll-like receptor 9 (TLR9) were observed in TP pigs than those of DLY pigs (*p* < 0.05), as were Interleukin 10 (IL-10) and zonular occludens 1 (ZO-1) (*p* < 0.01). Similar levels of bacterial richness and diversity were found in the jejunal microbiota of the TP and DLY pigs. However, the TP pigs showed a significantly different microbiome compared to DLY pigs at the genus level (ANOSIM; *p* < 0.05). *Pseudomonas, Stenotrophomonas, Phenylobacterium*, and *Sandaracinobacter* were enriched in DLY pigs (*p* < 0.05), while the *Lactobacillus* and *Solibacillus* had higher abundances in TP pigs than DLY pigs (*p* < 0.05). Tibetan pigs have “healthier” intestinal microbial communities than DLY pigs. Close relationships were found between jejunal immune performance and the differential bacteria, *Lactobacillus* can enhance porcine jejunal immunity, while *Stenotrophomonas* will have a negative impact on porcine gut immunity.

## Introduction

The gastrointestinal tract of mammals comprises approximately 500–1,000 microbes (1) that constitute complicated and dynamic microbial communities mainly consist of bacteria (2). Indeed, gut microbiota are concerned with the regular physiology of the gastrointestinal tract, including degrading indigestible carbohydrate like plant polysaccharides and producing diverse metabolites like short chain fatty acids (SCFAs) and vitamins (3). Besides metabolic advantages, commensal gut microbiota provide the host with functions that promoting immune responses, immune homeostasis and defending against pathogen invasion (4, 5). Furthermore, alterations in microbial community can affect immune system development and modulate immune mediators (6, 7). The microbiota of small intestine also have a considerable influence on diverse aspects of the host's physiology, including metabolism, immunity and endocrine (8).

The previous studies have suggested that genetic factor was broadly conductive to the difference of the intestinal microbial composition (9) and pig breeds strongly affect the bacterial structure at the suckling period (10). The Duroc × (Landrace × Yorkshire) (DLY) pig is a worldwide commercial pig breed by means of high breeding selection to pursue a faster growth rate at the detriment of decreasing the capacity of disease resistance. On the contrary, the Tibetan pig is a local pig breed that is found primarily in the Tibetan highlands and whose specific phenotype and physiology that have allowed them to accommodate the extreme environments (11).

However, very little information is available regarding the jejunal immunity and microbial structure in growing-period Tibetan pigs. Exploring how host genetics and pig breed affect intestinal microbial diversity and composition is of great importance in the understanding of the host health. Besides, searching the relationships between gut microbiota and immunity may help us solve health problems in pig industry. Therefore, the present study studied the jejunal immunity and microbial differences in Tibetan and DLY pigs and investigated the correlations between immune indices and relative abundance of differential microbiota.

## Materials and Methods

### Animals and Sample Collection

Twelve Tibetan pigs (4 months of age) and twelve DLY [Duroc × (Landrace × Yorkshire)] pigs (4 months of age) in the growth stage were selected and maintained in the same house with one pig per pen which equips with fully slatted floors, a feeder, and a nipple drinker. All pigs were housed at Yunnan Agricultural University and fed by the same NRC (NRC 2012) diet for 28 days (Table 1), then euthanized via exsanguination. Jejunal chyme samples were collected by advancing the lumenal contents into a cryotube. The jejunum samples (2–3 cm in middle section) were collected and fixed in 4% neutral paraformaldehyde fix solution. The treatment of the pigs was approved by the Institutional Animal Care and Use Committee of Yunnan Agricultural University (No. YNAU20201304).

**Table 1:** Basic diet compositions and nutritional levels.

**Items**	**Content (%)**	**Nutrient levels**	**Content**
Corn	42	Digestible energy (MJ/kg)	11.7
soybean meal	11.5	Crude protein	14
Highland barley flour	13	Ca	0.6
wheat bran	29.5	Total *P*	0.4
L-lyssine-HCl	0.31	Na	0.08
DL-methionine	0.01	Cl	0.07
L- threonine	0.03	Lysine	0.87
CaHPO4·2H2O	0.85	Methionine	0.24
NaCl	0.12	Threonine	0.54
Limestone	1.68	Tryptophan	0.16
Premix^a^	1	NDF	20
Total	100		

a *Premix supplied per kg diet: vitamin A, 7,500 IU; vitamin D3, 2,200 IU; vitamin E, 30 IU; vitamin K3, 2.6 mg; vitamin B1, 2.6 mg; vitamin B2, 7.2 mg; vitamin B6, 4.28 mg; vitamin B12, 27 μg; Fe, 120 mg; Zn, 50 mg; Cu, 14 mg; Mn, 50 mg; I, 0.25 mg; Se, 0.3 mg; niacin, 30.3 mg; pantothenic acid, 13.8 mg; biotin, 0.11 mg*.

### Hematoxylin-Eosin Staining and Immunohistochemistry

Each jejunal sample was cut into 4 μm thick slides and embedded in paraffin after fixation. And then three to four transverse slides from each sample were chosen to stain with hematoxylin and eosin. For immunohistochemical staining, slides were heated in microwaves at 750 W for 15 min in citrate buffer (pH 6.0) for antigen repairing after deparaffinization and rehydration. Following washing with phosphate-buffered saline (PBS), slides were treated for 25 min with 3% H_2_O_2_ at 20–22°C and in a photophobic environment to block endogenous peroxidase activity, washed with PBS, and trated for 30 min at 20–22°C with 3% bovine serum albumin to block. Then the slides were incubated with the primary antibody including rabbit anti-CD3 (Servicebio Technology, Wuhan, CN, #GB13014, 1:50), rabbit anti-MYD88, mouse anti-TLR4 and rabbit anti-TLR9 (#GB11269 and #GB11266, 1:100), rabbit anti-ZO-1 and rabbit anti-CD4 (#GB11195 and #GB13064-2, 1:200), rabbit anti-TLR2 (#GB11554, 1:500), rabbit anti-IL-1β and rabbit anti-IL-6 (#GB11113 and #GB11117, 1:800), and rabbit anti-MUC-2 (#GB11344, 1:1,000) diluted in PBS and at 4°C overnight. Next, the slides were washed with PBS and incubated for 50 min at 20–22°C with a secondary antibody HRP conjugated goat anti-rabbit IgG (#GB23303, 1:200). The immunohistochemical reaction was revealed for visual inspection using diaminobenzidine (DAB) as a chromogen (Vector Laboratories). Slides were further counterstained with hematoxylin and coverslipped. A digital microscopy scanner Pannoramic P250 (3DHISTECH Ltd., Budapest, Hungary) with a 20 × microscope objective was used to scan the immunostained slides. The corresponding whole-slide scanned images were evaluated using Pannoramic Viewer 1.15.2 (3DHISTECH Ltd., Budapest, Hungary). The final immunohistochemistry results were expressed as H-score as reported by Yang et al. (12).

### DNA Extraction and Amplicon Sequencing

Following the manufacturer's instructions, total microbial DNA extraction of jejunal contents sample was carried out using the HiPure Stool DNA Kits (Magen, Guangzhou, China). Amplicon pooled libraries for sequencing on an Illumina HiSeq 2500 (Illumina, San Diego, CA, USA) were prepared using primers 341F (5′-CCTACGGGNGGCWGCAG-3′) and 806R (5′-GGACTACHVGGGTATCTAAT-3′) targeted across the hypervariable V3-V4 regions of the 16S rRNA gene. The amplification was performed in the following steps: initial denaturation at 95°C for 2 min, followed by 27 cycles (98°C−10 s, 62°C−30 s, 68°C−30 s) and a final extension at 68°C for 10 min.

### Bioinformatics Analyses

FASTP (https://github.com/OpenGene/fastp) was used to filter the raw sequences, and then with a minimal overlap of 10 bp and mismatch error ratio of 2%, paired-end reads were assembled as raw tags through FLSAH (version 1.2.11). QIIME (version 1.9.1) pipeline was used to filter noisy sequences of raw tags to obtain high-quality clean tags. Subsequently, with a similarity ≥97%, UPARSE (http://drive5.com/uparse/) was used to cluster the tags into OTUs (operational taxonomic units), while all chimeric tags were removed using Userach (version 7.0). Each representative sequence was assigned into organisms by RDP (Ribosomal Database Project) classifier (version 2.2) with a confidence threshold value of 0.8–1. Alpha diversity of observed species, Good's coverage, Chao1, Shannon, Simpson, PD whole tree, and beta diversity of principal component analysis (PCA) was determined using QIIME software (version 1.9.1). Based on high-quality reads, functional categories of KEGG ortholog were predicted by Tax4Fun (version 1.0).

### Statistical Analysis

The analysis of intestinal morphology and immune performance results was calculated by Welch's *t*-test using the SPSS 22.0 software. The Wilcoxon rank test was used to compute differential functional analysis among groups in R project (version 3.4.1). Correlations between variables were calculated using the Spearman rank correlation in GraphPad Prism 7.0.

## Results

### Intestinal Morphology and Immune Performance

In the jejunum, TP pigs had higher villus height and less deeper crypt depth than DLY pigs (*p* < 0.05). And TP pigs tended to have higher villus height to crypt depth ratio than DLY pigs (*p* = 0.162) (Table 2).

**Table 2:** Comparison of jejunal intestinal morphology of growing DLY and TP pigs.

	**TP**	**DLY**	***p***
Villus height, μm	516.10 ± 1.9^a^	472.85 ± 12.63^b^	0.028
Crypt deepth, μm	152.13 ± 2.03^B^	191.18 ± 6.33^A^	0.004
Villus height/Crypt depth	3.43 ± 0.01	1.65 ± 0.82	0.162

^a, b^*Means in the same row with different superscripts are significantly different (p < 0.05)*.

^A, B^*Means in the same row with different superscripts are significantly different (p < 0.01)*.

*n = 12 per treatment group (Mean ± SEM)*.

As shown in Table 3, at the protein level, the cluster of differentiation 4 (CD4+) and Toll-like receptor 9 (TLR9) were significantly higher in TP pigs than in DLY pigs (*p* < 0.05), as were the interleukin 10 (IL-10) and zonular occludens 1 (ZO-1) contents (*p* < 0.01).

**Table 3:** Comparison of jejunal mucosal immunity related indices of growing TP and DLY pigs.

	**TP**	**DLY**	***p***
IL-6	35.8 ± 18.84	17.67 ± 9.37	0.437
CD3^+^	23.92 ± 1.36	24.09 ± 3.13	0.963
CD4^+^	51.04 ± 1.97^a^	42.4 ± 1.65^b^	0.028
IL-10	19.67 ± 1.28^A^	4.2 ± 1.22^B^	0.001
TLR2	2.41 ± 0.5	1.42 ± 0.3	0.165
TLR4	1.53 ± 0.05	2.42 ± 0.63	0.292
TLR9	48.73 ± 6.89^a^	25.72 ± 3.74^b^	0.043
MYD88	34.78 ± 3.17	33.03 ± 1.07	0.629
MUC2	23.54 ± 4.46	14.25 ± 1.2	0.115
ZO-1	16.11 ± 1.02^A^	5.65 ± 1.58^B^	0.008
IL-1β	45.19 ± 5.82	17.14 ± 8.28	0.050

^a, b^*Means in the same row with different superscripts are significantly different (p < 0.05)*.

^A, B^*Means in the same row with different superscripts are significantly different (p < 0.01)*.

*>n = 12 per treatment group (Mean ± SEM)*.

### Microbial Diversity in Jejunum of TP and DLY Groups

**Figure 1:**
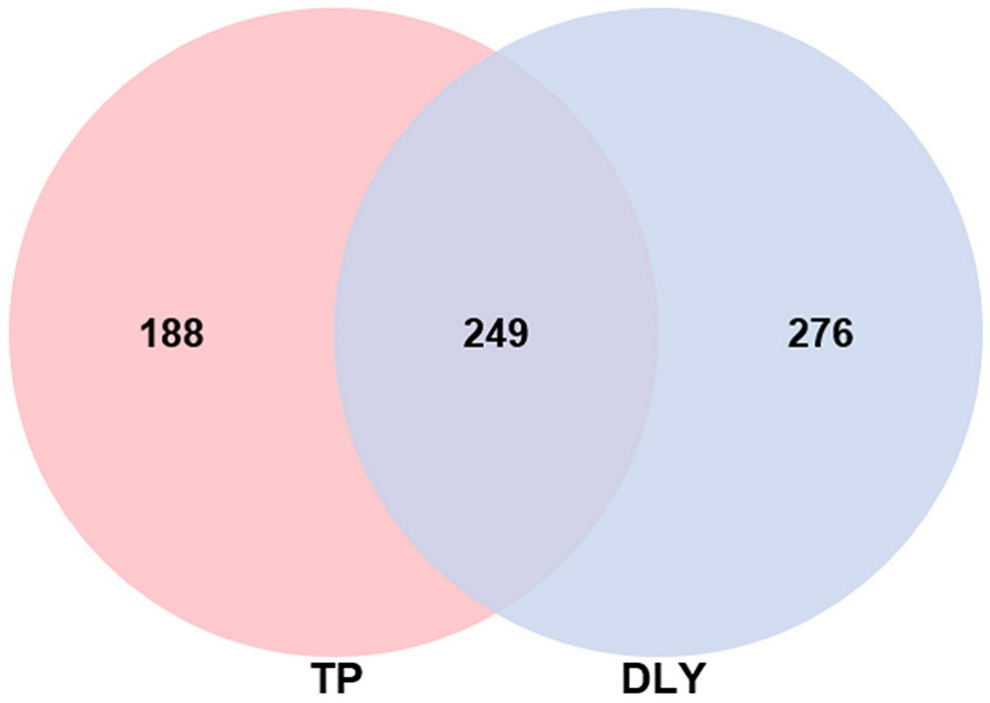
Venn diagram of jejunum chyme of growing TP pigs and DLY pigs.

We obtained 2,925,307 clean tags from the samples by using the IlluminaHiSeq2500 platform. Of these, 2,881,333 effective tags with an average length of 421 bp were selected after the quality control and filtering. This yielded an average of 120,055 effective reads per sample. The average effective tags to clean tags ratio was 94.68% (Table 4). We identified a total of 6,156 operational taxonomic units (OTUs) at a 97% similarity level. A Venn diagram (Figure 1) shows 249 OTUs are shared between TP and DLY groups, along with 188 and 276 unique OTUs in the TP and DLY groups, respectively.

**Table 4:** Original data sheet of jejunal microorganism 16S rRNA gene sequencing.

**Sample**	**Clean**	**Effective**	**Effective**	**Average**	**OTUs**
**name**	**tags**	**tags**	**ratio (%)**	**length**	
TP-1	121090	118111	93.11	462.63	299
TP-2	123520	111061	86.39	463.2	278
TP-3	115562	113875	94.88	465.56	171
TP-4	116106	114789	95.16	465.63	203
TP-5	126511	123861	94.5	461.94	227
TP-6	122806	121521	95.43	463.91	227
TP-7	118432	117789	95.15	464.37	322
TP-8	134376	133146	97.79	459.73	246
TP-9	129811	128498	95.65	460.13	303
TP-10	130802	129822	95.44	464.1	260
TP-11	111466	109894	94.64	464.87	198
TP-12	120355	118812	94.99	465.04	217
DLY-1	129512	128254	95.01	461.37	312
DLY-2	123461	122487	94.42	456.09	278
DLY-3	117354	116860	95.56	465.5	188
DLY-4	114414	111688	91.77	461.78	250
DLY-5	121283	120819	94.64	465.07	218
DLY-6	116354	114749	93.9	464.11	218
DLY-7	114914	114104	94.55	463.43	241
DLY-8	122730	121666	97.38	460.21	239
DLY-9	126704	125785	94.86	463.43	266
DLY-10	122873	121516	94.93	465.64	199
DLY-11	124004	123088	95.05	460.49	281
DLY-12	120867	119147	97.2	458.67	269

Good's coverage for the two groups was >99.5%, indicating a satisfactory sequencing depth of the gut microbiota (Table 5). Alpha diversity indices were not significantly different between TP and DLY pigs (*p* > 0.05). For example, the Shannon index (*p* = 0.281) and simpson (*p* = 0.523) index of TP pigs were lower than those of DLY pigs.

**Table 5:** Comparison of alpha diversity estimation of the 16S rRNA gene libraries for TP and DLY pigs at 97% similarity.

**Group**		**TP**	**DLY**	***p***
Richness	Goods coverage	0.9999	0.9999	0.862
estimation	chao 1	308.78 ± 13.35	311.98 ± 12.70	0.864
Diversity	observed_species	245.92 ± 13.59	246.58 ± 10.57	0.969
index	Shannon	2.60 ± 0.29	3.19 ± 0.44	0.281
	Simpson	0.62 ± 0.06	0.68 ± 0.07	0.523

**Figure 2:**
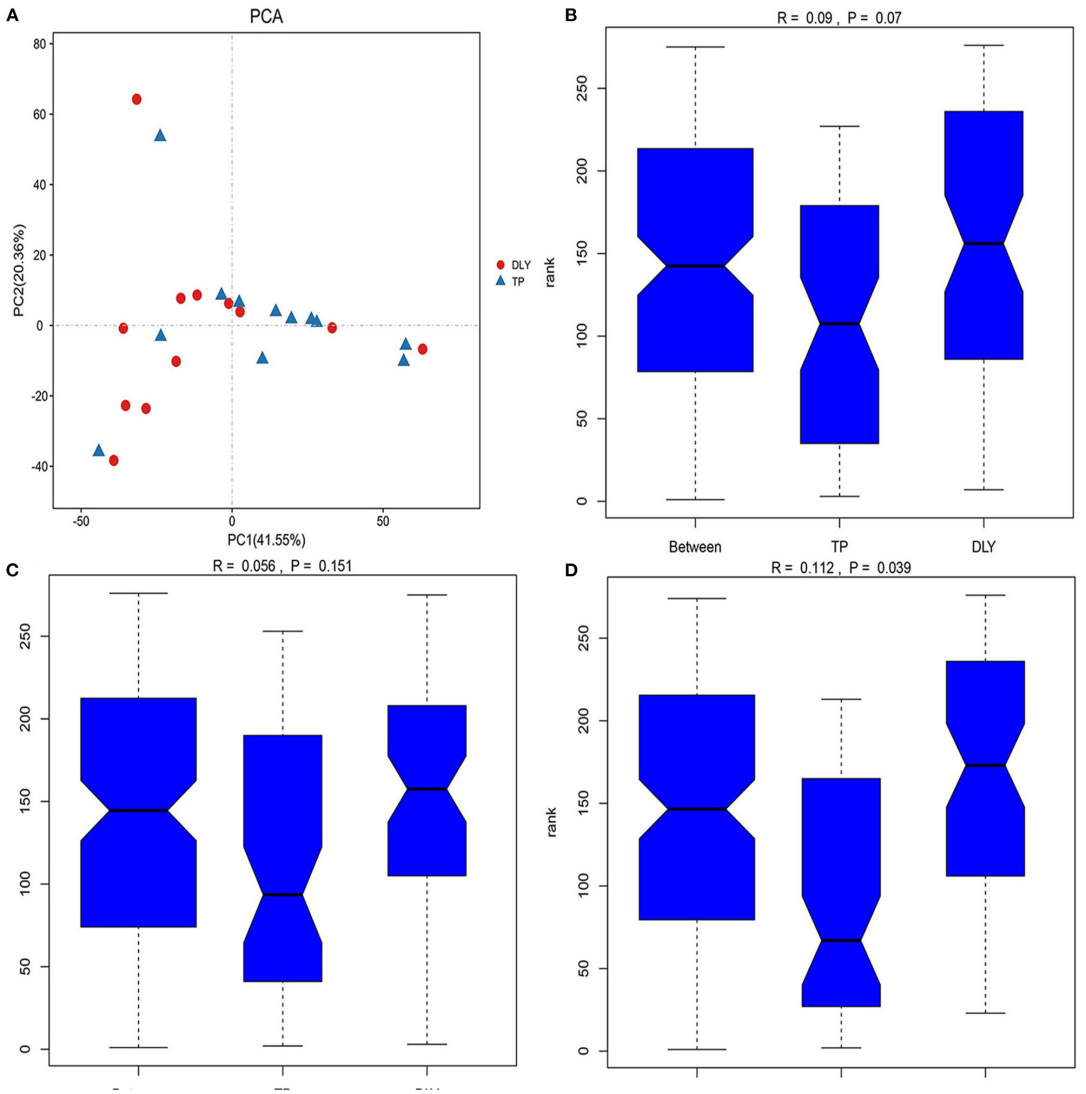
Beta-diversity analysis of jejunal microbiota between TP and DLY pigs. **(A)** PCA analysis; **(B)** ANOSIM analysis at the OTU level; **(C)** ANOSIM analysis at the phylum level; **(D)** ANOSIM analysis at the genus level.

The principal component analysis (PCA) score plot (Figure 2A) from sequences at the OTU level showed that the microbial composition of TP pigs was like that of DLY pigs, and as were the analysis of similarity (ANOSIM) at the OTU and phylum levels (*p* > 0.05) (Figures 2B,C). However, the TP pigs showed a significant different microbiome compared to DLY pigs at the genus level, as shown in Figure 2D (ANOSIM; *p* < 0.05).

### Jejunal Microbial Composition and Differences Between TP and DLY Groups

**Figure 3:**
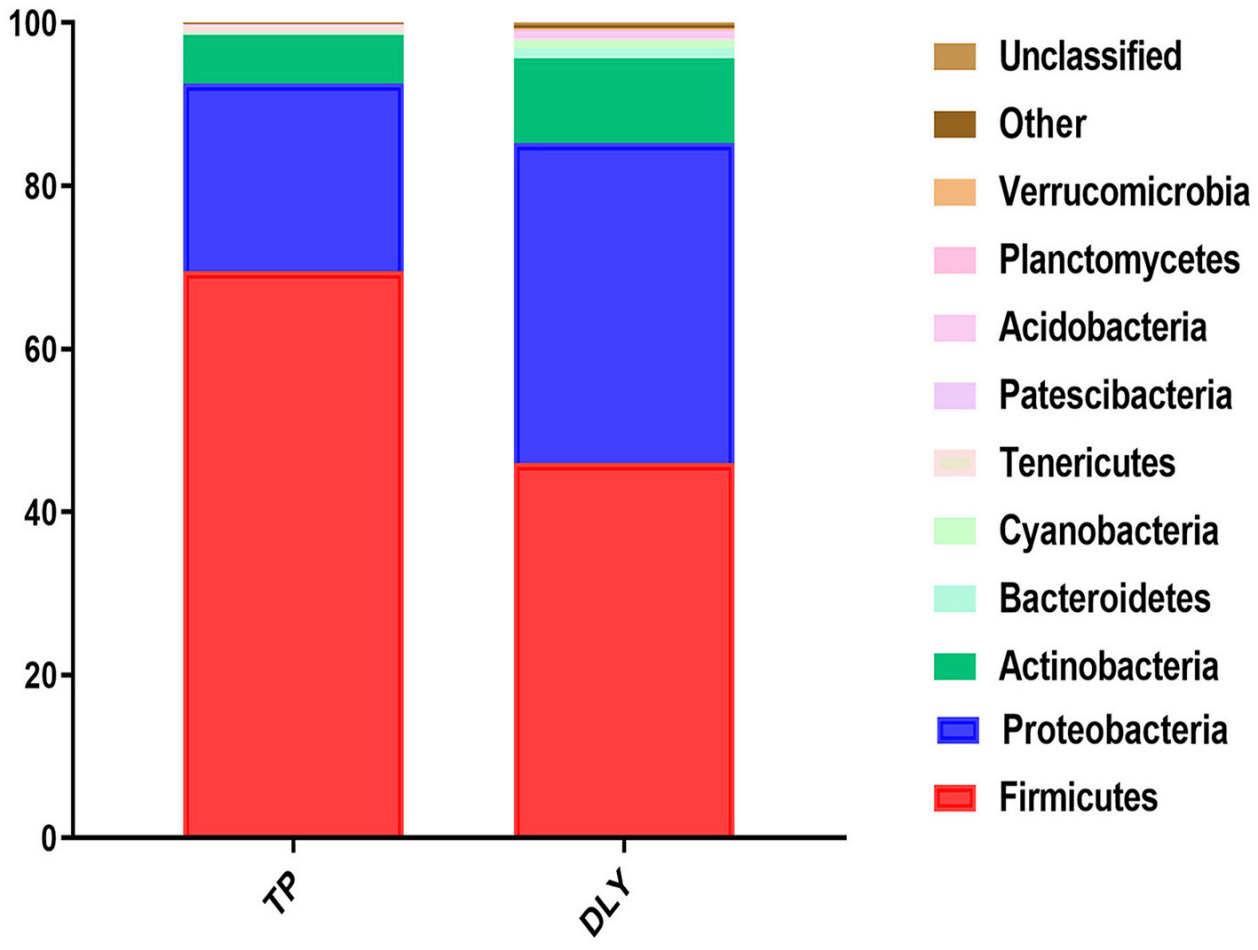
Relative jejunal abundance at the phylum level in TP and DLY pigs.

The predominant phyla were *Firmicutes, Proteobacteria, Actinobacteria*, and *Bacteroidetes* (Figure 3). The relative abundance of *Tenericutes* in TP (0.76%) pigs was significantly higher than in DLY (0.24%) pigs (*p* < 0.05). The TP pigs displayed a 23.53% increase in the relative abundances of *Firmicutes* compared with DLY pigs (*p* = 0.07), while the relative abundance of *Proteobacteria* (*p* = 0.24), *Actinobacteria* (*p* = 0.25), *Bacteroidetes* (*p* = 0.06), and *Cyanobacteria* (*p* = 0.16) tended to be lower in the TP pigs than in the DLY pigs (Table 6).

**Table 6:** Analysis of the jejunal composition and differences at the phylum level of growing DLY and TP pigs.

**Phylum**	**TP**	**DLY**	***p***
Firmicutes	69.53 ± 7.91	46.00 ± 9.14	0.07
Proteobacteria	23.03 ± 8.40	39.27 ± 10.12	0.24
Actinobacteria	5.91 ± 1.93	10.38 ± 3.25	0.25
Bacteroidetes	0.13 ± 0.04	1.22 ± 0.53	0.06
Cyanobacteria	0.31 ± 0.08	0.96 ± 0.44	0.16
Tenericutes	0.76 ± 0.43^a^	0.24 ± 0.17^b^	0.04

^a, b^*Means in the same row with different superscripts are significantly different (p < 0.05)*.

*Means in the same row with different superscripts are significantly different (p < 0.01)*.

*n = 12 per treatment group (Mean ± SEM)*.

**Figure 4:**
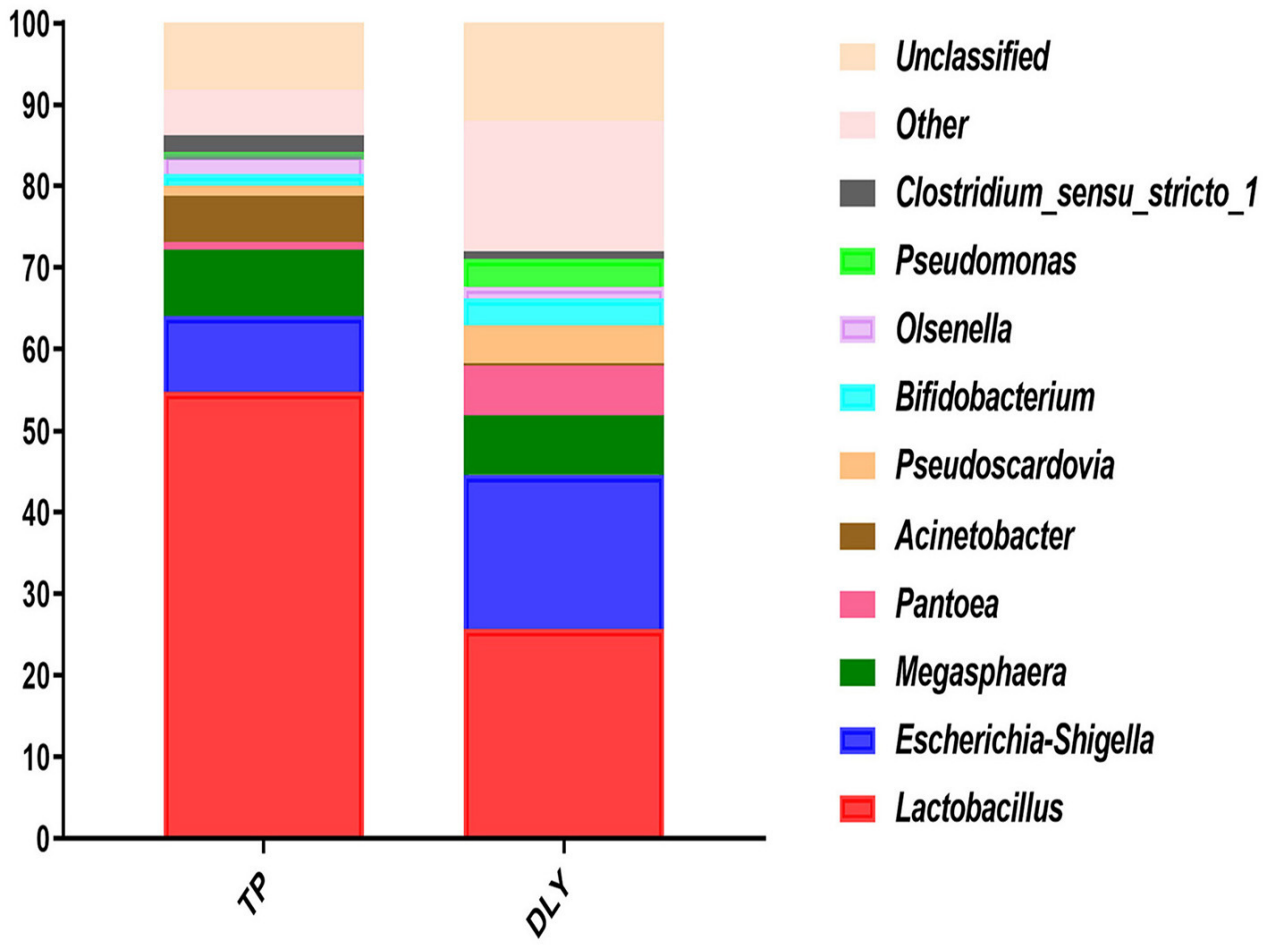
Relative jejunal abundance at the genus level for TP and DLY pigs.

At the genus level, the most predominant was *Lactobacillus*, followed by *Escherichia-Shigella, Megasphaera, Pantoea, Acinetobacter, Pseudoscardovia, Pseudomonas, Bifidobacterium, Streptococcus, Olsenella*, and *Clostridium_sensu_stricto_1* (Figure 4). As shown in Table 7, TP pigs manifested an increase of 29.05 and 0.47% increase in the relative abundances of *Lactobacillus* and *Solibacillus* compared with DLY pigs, while the relative abundances of *Pseudomonas, Stenotrophomonas, Phenylobacterium*, and *Sandaracinobacter* in TP pigs were significantly lower than those in DLY pigs (*p* < 0.05).

**Table 7:** The differential jejunal microbiota at the genus level for TP and DLY pigs.

	**TP**	**DLY**	***p***
*Lactobacillus*	54.74 ± 8.76^a^	25.69 ± 8.54^b^	0.03
*Solibacillus*	0.48 ± 0.31^a^	0.0077 ± 0.00^b^	0.02
*Pseudomonas*	0.12 ± 0.04^b^	3.44 ± 2.37^a^	0.02
*Stenotrophomonas*	0.05 ± 0.03^b^	0.29 ± 0.11^a^	0.02
*Phenylobacterium*	0^b^	0.13 ± 0.11^a^	0.03
*Sandaracinobacter*	0^b^	0.02 ± 0.02^a^	0.02

^a, b^*Means in the same row with different superscripts are significantly different (p < 0.05)*.

*Means in the same row with different superscripts are significantly different (p < 0.01)*.

*n = 12 per treatment group (Mean ± SEM)*.

### Correlation Analysis of the Gut Microbiota and Immunity Factors

**Figure 5:**
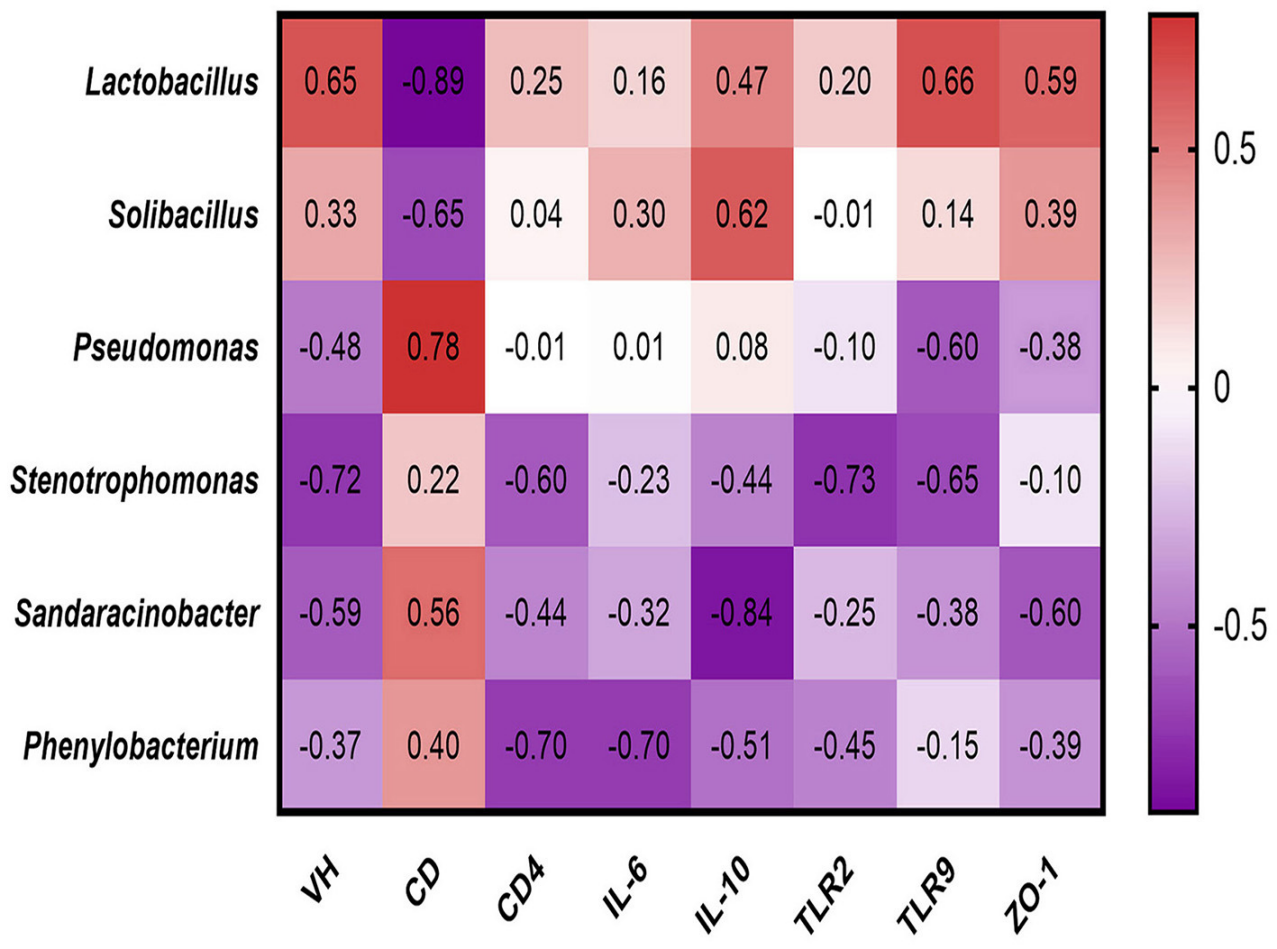
Spearman's rank correlations between differential jejunal microbiota and immune performance.

Spearman's rank correlations (Figure 5) between the jejunal morphology phenotype, immune performance and relative abundance of differential microbiota were assessed, the relative abundance of *Lactobacillus* positively correlated with villus height (*R* = 0.65, *p* = 0.049), TLR9 expression at the protein level (*R* = 0.66, *p* = 0.044) negatively correlated with crypt depth (*R* = −0.89, *p* = 0.0011). The relative abundance of *Solibacillus* negatively correlated with crypt depth (*R* = −0.65, *p* = 0.049). The relative abundance of *Pseudomonas* positively correlated crypt depth (*R* = 0.78, *p* = 0.011). The relative abundance of *Stenotrophomonas* negatively correlated with villus height (*R* = −0.72, *p* = 0.024), TLR2 (*R* = −0.73, *p* = 0.021) and TLR9 expression at the protein level (*R* = −0.65, *p* = 0.047). The relative abundance of *Sandaracinobacter* negatively correlated with IL-10 (*R* = −0.84, *p* = 0.040). The relative abundance of *Phenylobacterium* negatively correlated with CD4+(*R* = −0.70, *p* = 0.022) and IL-6 expression at the protein level (*R* = −0.70, *p* = 0.022).

### The Predictive Microbial Functions of the Jejunum in TP and DLY Pigs

**Figure 6:**
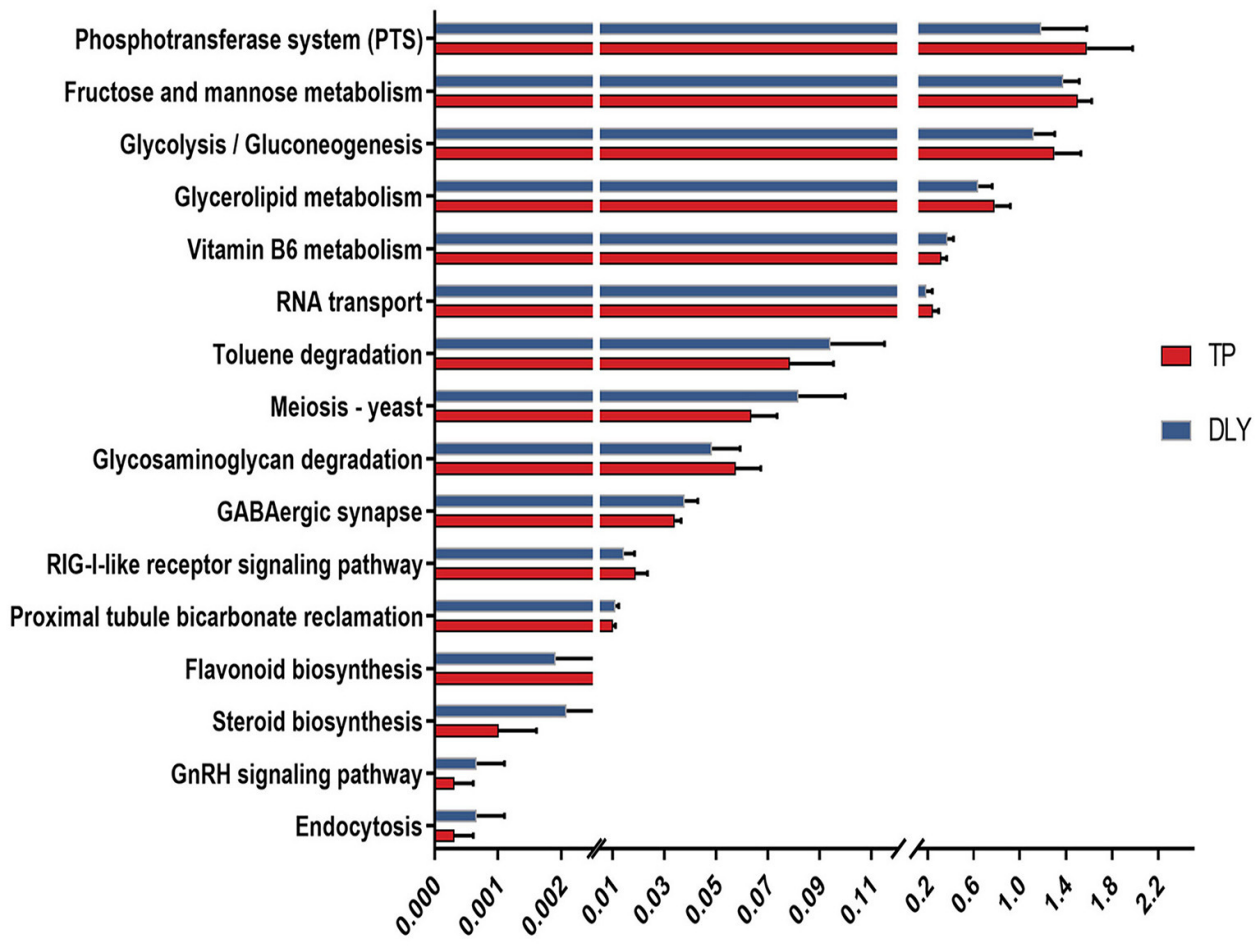
Differential KEGG pathway analysis based on Tax4Fun functional prediction.

According to the Tax4Fun-based functional prediction, 16 differential KEGG pathways were found of the jejunal microbiota between the TP and DLY pigs (Figure 6). The pathways enriched in the TP pigs were mainly related to fructose and mannose metabolism, phosphotransferase system (PTS), glycerolipid metabolism, glycolysis/gluconeogenesis, RNA transport, glycosaminoglycan degradation, RIG-I-like receptor signaling pathway and flavonoid biosynthesis (*p* < 0.05). The pathways enriched in the DLY pigs were mainly related to Vitamin B6 metabolism, Toluene degradation, Meiosis – yeast, GABAergic synapse, Proximal tubule bicarbonate reclamation, Steroid biosynthesis, GnRH signaling pathway and Endocytosis (*p* < 0.05).

## Discussion

It is generally considered that the gastrointestinal tract is the largest immune organ in mammals and involves in modulating immunological homeostasis (13). In our study, the levels of jejunal IL-10, CD4+, TLR9, and ZO-1 were higher in Tibetan pigs than in DLY pigs. IL-10 is a cytokine with widely anti-inflammatory characteristics as well as a vital regulatory role of CD4+ T lymphocytes (14). CD4+ T lymphocytes are reported to be importantly involved in defending mammals against pathogens and are necessary for boosting cytokine production (15). ZO-1 is the principal component of the functional and constructional organization of tight junctions related to epithelial integrity (16). Porcine toll-like receptors are thought to be the frontline of pathogen monitoring and may be associated with disease tolerance (17). Our results indicate that Tibetan pigs may have better anti-inflammatory characteristics and intestinal barrier function than DLY pigs. Consistent with our research, the research of Gao et al. showed that Jinhua pigs showed lower immune activation and diarrhea frequency than Landrace pigs when challenging with an enterotoxigenic E. coli (ETEC) K88 species (18). Albin et al.' s research shows that resistance (decreasing conductance) increased in intestines of Yorkshire pigs in response to lipopolysaccharide (LPS), whereas resistance in Meishan intestines was unchanged with LPS (19). This indicates that the gut barrier functions are likely enhanced in the traditional pig breeds comparing that in the Yorkshire pigs. Furthermore, a previous study found that innate immunity in Tibetan pigs were stronger than Yorkshire pigs (20). These results indicate that TP pigs may have better anti-inflammatory properties and gut barrier functions than DLY pigs.

Consistent with our research, some researchers have found that small intestine of weanling piglets had the highest populations of *Firmicutes* and moderate levels of *Proteobacteria* and *Actinobacteria* (21). The most dominant genus was *Lactobacillus*, which is related to the fact that the small intestine is abounded in mono- and di-saccharides along with amino acids, supporting the proliferation of *Lactobacillus* (22). Recent studies have indicated that the pig breed and host genetics play fundamental roles in the microbial profiles of gastrointestinal tract (23). Our results show that the relative abundances of *Lactobacillus* and *Solibacillus* were higher in TP pigs than in DLY pigs, while *Pseudomonas, Stenotrophomonas, Phenylobacterium*, and *Sandaracinobacter* were lower in TP pigs than in DLY pigs, which indicated that the intestine microbial composition of different pig breeds was different. Consistent with our research, Pajarillo et al. found that feces of Duroc pigs had higher relative abundances of fecal *Phascolarctobacterium, Catenibacterium*, and *Subdoligranulum* than those in Landrace and Yorkshire pigs (10). Compared with Rongchang (RP) and TP pigs, Yorkshire pigs (YP) had a lower ratio of *Firmicutes* and *Bacteroidetes*, while higher abundances of *Spirochaetes* were found in TP in comparison with RP and YP (24). It is indicated that the host genetic factors have an influence on the structure and abundance of intestinal microbiota at the genus level. In addition, *Lactobacillus* are reported as probiotics in virtue of their anti-inflammatory ability (25), presence of three species of *Solibacillus* have been reported, of which *Solibacillus silvestris* can produce antineoplastic biomolecule (26). On the other hand, *Stenotrophomonas* species are multi-resistant bacteria with ability to cause opportunistic infections (27), and *Pseudomonas* has been reported as a known pathogen of mastitis microbiota in ruminants (28). Consequently, the intestinal microflora of TP pigs are more probably “healthier” than DLY pigs.

It is well-known that the intestinal microbiota composition influenced immune system development and modulated immune mediators. In this study, we found that *Lactobacillus* was positively correlated with IL-10 whereas the potential harmful *Stenotrophomonas* in this study were negatively with TLR2 and TLR9. The members within the genus *Lactobacillus* are reported to maintain the intestinal ecological equilibrium through avoiding the invasion of pathogens, modulating immunity, and enhancing mucosal barrier integrity (29). For example, treatment of porcine intestinal IPEC-1 epithelial cells with *Lactobacillus sobrius* repealed the upregulation in IL-1β and IL-8 levels and induced an increase of IL-10 (30). *Stenotrophomonas* is an multidrug-resistant global opportunistic pathogen, which is related to the increase of human and animal infections in recent years (31–33). In addition, researches shows that the reduction of pathogenic bacteria is helpful to enhance the immune performance of the body (32, 33). These results indicates that *Lactobacillus* play a vital role in enhancing porcine jejunal immunity while *Stenotrophomonas* have a negative effect on porcine jejunal immunity.

## Conclusions

In summary, the present study revealed that the jejunal microbial diversity did not differ between TP pigs and DLY pigs, while jejunal immunity and microbial composition were different. TP pigs have better anti-inflammatory properties and gut barrier functions than DLY pigs, and TP pigs enhance the jejunal immunity through *Lactobacillus*, while *Stenotrophomonas* in the jejunum of DLY pigs has a negative impact on its jejunal immunity.

## Data Availability Statement

The datasets presented in this study can be found in online repositories. The names of the repository/repositories and accession number(s) can be found in the article/supplementary material.

## Ethics Statement

The animal study was reviewed and approved by all animal research was approved by the Ethics Committee of Yunnan Agricultural University (Approval No: YNAU20201304). Written informed consent was obtained from the owners for the participation of their animals in this study.

## Author Contributions

HP designed the experiments. HP, YY, YL, YX, and LY performed the experiments. YY and YL analyzed the data and wrote the manuscript. SQ and HP revised this manuscript. All authors contributed to the article and approved the submitted version.

## Funding

This study was supported by Nature Science Foundation of China (Nos. U1802234 and 32160795).

## Conflict of Interest

The authors declare that the research was conducted in the absence of any commercial or financial relationships that could be construed as a potential conflict of interest.

## Publisher's Note

All claims expressed in this article are solely those of the authors and do not necessarily represent those of their affiliated organizations, or those of the publisher, the editors and the reviewers. Any product that may be evaluated in this article, or claim that may be made by its manufacturer, is not guaranteed or endorsed by the publisher.
